# Supplementary material for: Characterization of the complete mitochondrial genome of the Sunda stink-badger (Mydaus javanensis) from the island of Borneo
Source: PeerJ. 2024 Oct 14;12:e18190. doi: 10.7717/peerj.18190 (PMC11485102; doi:10.7717/peerj.18190)
Supplement: Supplemental Information 6 [file peerj-12-18190-s006.docx]

**Rebuttal Letter Supporting Author Addition (Ranjita Subramaniam)**

Here, I would like to address the concerns regarding the addition of Ranjita Subramaniam as an author on the revised version of the manuscript, and to provide detailed reasons for this inclusion.

During the revision process, Ranjita Subramaniam undertook several significant tasks that warrant authorship. Her contributions are as follows:

1. **Data Analysis on Control Region of Mitochondria**:
   - **Organization of the Control Region**:

Ranjita conducted research on the mitochondrial control region of mephitids. Through nucleotide sequence analysis, she discovered that the organization of mephitids closely resembles that of multiple reported species. She found that the basic structures were similar to those of mustelids and some other mephitids. Ranjita extracted and aligned the sequences of mephitids and analyzed the tandem repeats concurrently.

- - **Comparative Study**: She analysed the overall data on similarities and differences in the control region among mephitids.
  - **Figures and Tables**: She created all associated figures and tables to present the findings clearly.
  - **Results and Discussion**: Ranjita wrote the sections detailing the results of the control region analysis and discussed the implications of the findings.

1. **Phylogenetic Analyses**:
   - **Reconstruction and Analysis**:

Ranjita had reanalysed the availability of mitogenomes and cytochrome b of the mephitids to be included for the reconstruction of phylogenetic trees, for the respective datasets. Ranjita had reperformed concatenation of the 13 PCGs to precisely locate the positions of each gene for the purpose of running partition finding. Followingly, she had conducted the phylogenetic analyses of the datasets through ML and BI approaches.

- - **Figures and Tables**: Ranjita created all associated figures and tables for presenting the phylogenetic relationships of *Mydaus* and the related species.
  - **Results and Discussion**: She authored the results and discussion sections for thse analyses.

1. **Manuscript Revision**:
   - **Rewriting and Improvement**: Ranjita amended the entire manuscript as per the revision process, improving clarity, coherence, and scientific rigor. She made substantial changes to the text, enhancing the overall quality of the manuscript. She had made all the changes present in the revised manuscript from Introduction, Materials and Methods, Results and Discussion. She had freshly authored the Results and Discusion for mitochondrial control region and phylogenetic analyses as well as Conclusion.
   - **New Datasets and Findings**: Ranjita Subramaniam had generated new datasets and findings, which are integral to the revised manuscript.

Given the extent of Ranjita’s contributions, her inclusion as an author is justified.

**These efforts were not part of the initial submission but were conducted during the revision phase. As such, her name was not found in the initial submission. She had worked on the manuscript from the month of May to June, which was the timeline when Ranjita had performed the entire revision process.**

**All current co-authors have agreed to this change.**

**Additionally, in the revised manuscript, I have used the commenting functionality to clearly indicate which parts of the new text resulted from her contributions. This ensures transparency and aligns with your guidelines.**

I appreciate your understanding and consideration of Ranjita’s authorship based on the significant contributions outlined above.
